# Botulinum Toxin for Lip Inversion in Gummy Smile

**DOI:** 10.7759/cureus.107571

**Published:** 2026-04-23

**Authors:** Sandeep Arora, Gulhima Arora

**Affiliations:** 1 Dermatology, Mehektagul Dermaclinic, New Delhi, IND; 2 Dermatology, Apollo Spectra Hospitals, New Delhi, IND

**Keywords:** botulinum toxin injection, gum line show, gummy smile correction, lip inversion correction, yonsei point

## Abstract

A gummy smile results from varied factors, including excessive muscle activity, maxillary bone variations, dental malformations, or smaller lip volume. Botulinum toxin is used to correct a gummy smile when there is excessive vertical upper lip mobility. Lip inversion can accompany a gummy smile. We report a case of lip inversion accompanying a gummy smile corrected with botulinum toxin. This simple procedure can be instituted at the stage of gummy smile correction or upon follow-up for its correction, and can obviate the need for overdosing and the use of lip fillers.

## Introduction

Gingival exposure of 3 mm or more during a fully posed smile is defined as excessive gingival display (EGD). It affects approximately 10-14% of young adults and is more commonly reported in women [1,2]. Its social significance is considerable; affected individuals frequently report self-consciousness about their smile and curtail expressive facial behaviour [3]. The underlying cause may vary from hypercontractility of the upper lip elevators to vertical maxillary excess, short crowns, or dentoalveolar protrusion. Treatment selection depends on the accurate aetiological diagnosis [2,4].

Among the lip elevators, the levator labii superioris alaeque nasi (LLSAN), levator labii superioris (LLS), and zygomaticus minor (ZMi) bear principal responsibility for upward lip excursion during smiling. Hwang et al. demonstrated through the cadaveric dissection of 50 hemi-faces that these three muscles converge on a small triangular region lateral to the alar base, the centre of which they designated the Yonsei point [5]. This anatomical convergence makes it a logical single-site injection target, and subsequent clinical data supported its reproducibility across diverse smile patterns [6,7].

Less attention has been paid to lip inversion as a companion finding. During smiling, the superior orbicularis oris exerts an inward and downward force on the lip margin. Usually, this is counterbalanced by the elevator muscles. When lip inversion is pronounced, the vermilion disappears from view, producing a thin, taut upper lip which many find equally or more bothersome than gingival exposure itself. The lip flip addresses this directly: small doses of BoNT-A (botulinum neurotoxin type A) placed into the superior orbicularis oris fibres selectively reduce the inward vector without disrupting overall sphincteric competence, allowing passive eversion of the vermilion [8,9].

The case described here draws attention to a gap that persists in routine EGD assessment. Lip inversion when visible at baseline or unmasked by toxin-induced elevator relaxation can be managed with the same botulinum toxin, avoiding the reflexive escalation to higher toxin doses or filler augmentation that can otherwise complicate gummy smile management.

## Case presentation

**Figure 1:**
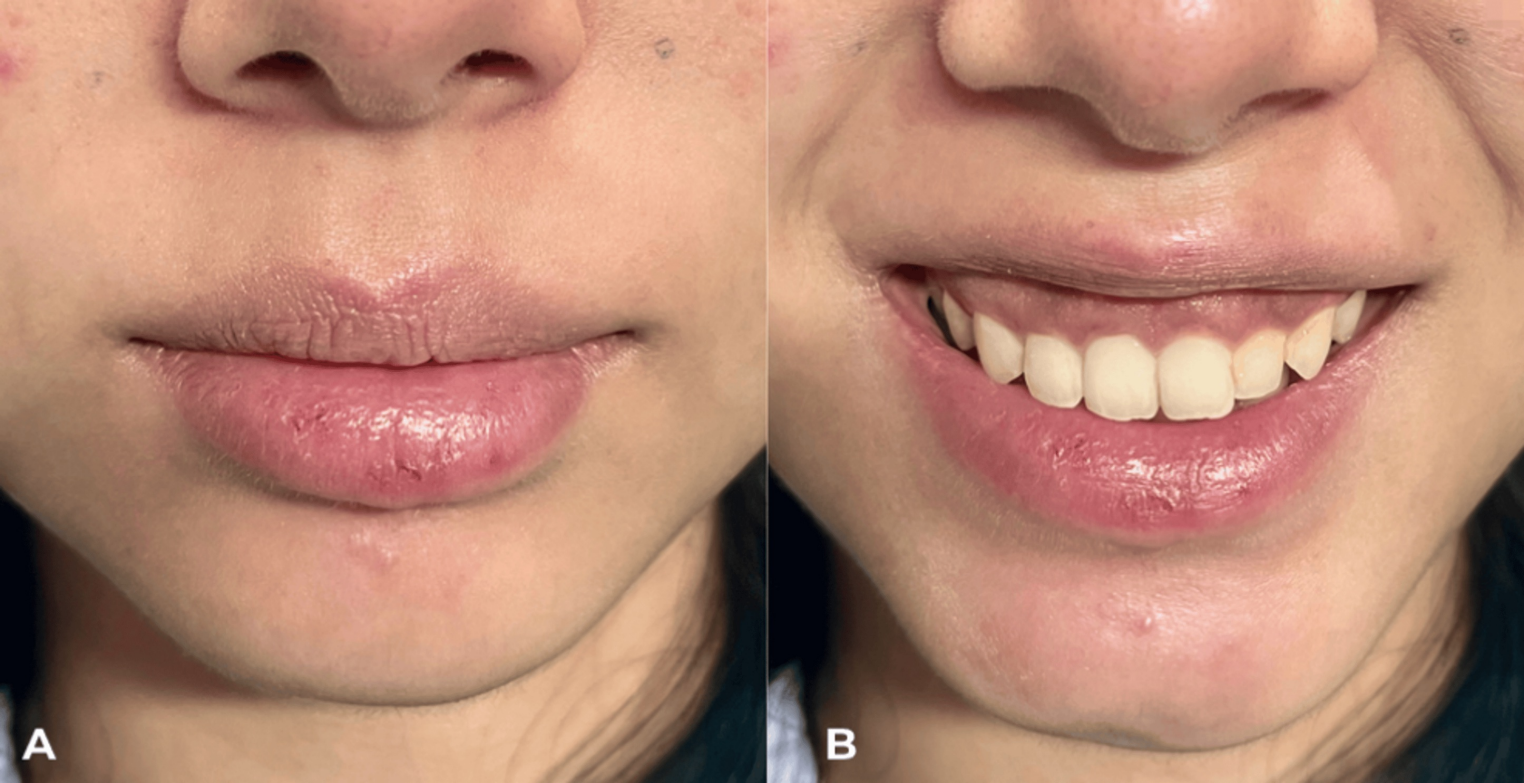
(A) Lips pretreatment. (B) Gummy smile at baseline with injection points (2 U at each point)

A 24-year-old woman with no prior facial injectable treatments and no relevant medical history presented for gummy smile correction. Clinical assessment included observation at rest, during a relaxed conversational smile, and at a maximal smile. Dentition was intact and well-positioned; there was no clinical evidence of vertical maxillary excess, altered passive eruption, or significant alveolar protrusion. Resting lip volume was judged appropriate for her facial proportions (Figure 1A). The patient had a cuspid-dominant smile pattern [10] with 4-5 mm of anterior gingival exposure in the premaxillary segment (Figure 1B). Subtle upper lip inversion was noted during maximal smile but was considered a secondary finding at baseline, with the gingival display constituting the primary complaint.

At the first treatment visit, onabotulinumtoxinA (Stunnox®, reconstituted with 1.25 ml preservative-free 0.9% NaCl to 40 U/ml) was injected intramuscularly at the Yonsei point bilaterally, 2 U per side (total 4 U), using a 31-gauge needle. No adverse events occurred.

Upon follow-up on the 10th day, the gingival exposure had resolved completely. However, with the elevator muscles partially attenuated, the relative dominance of the superior orbicularis oris became apparent: the upper lip rolled inward during smiling, effacing the vermilion and producing a flattened contour at odds with the patient's resting lip morphology. The inversion previously subordinate to the gingival display had been unmasked by correction of the primary problem.

**Figure 2:**
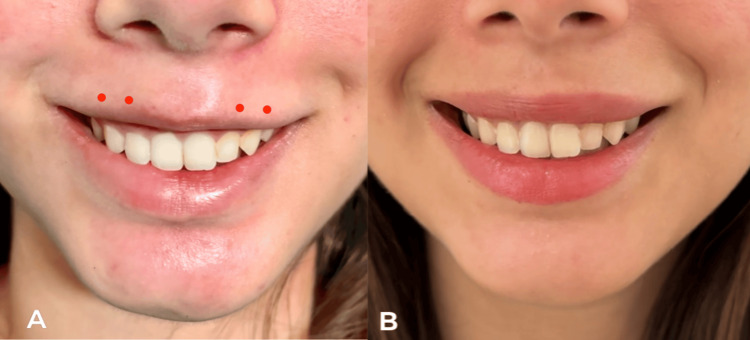
(A) Corrected gummy smile with persistent lip inversion showing injection points with units for lip inversion (0.5 U at each point). (B) Corrected gummy smile and lip inversion

A lip flip was performed with 0.5 U of Stunnox® injected intramuscularly at each of four points along the superior vermilion border for a further 2 U (Figure 2A). Injections were placed 2-3 mm above the mucocutaneous junction, within the muscle belly, to selectively weaken the superior sphincteric fibres [8,9,11]. Two weeks after the lip flip, the patient showed full resolution of both the gummy smile and lip inversion (Figure 2B). The vermilion show was restored, and the smile contour was harmonious with the dentition and perioral soft tissue. The patient reported complete satisfaction. There were no adverse events. Total BoNT-A used across both sessions was 6 U. No filler was administered. The patient was counselled that re-treatment would be expected at four to six months as muscular activity returns.

## Discussion

Gummy smile correction, followed by the emergence of lip inversion, reflects a clinically important dynamic that is under-recognised in standard EGD protocols. The lip elevator complex and the orbicularis oris act in opposition during smiling; selectively relaxing one alters the equilibrium and may shift attention to the other. Anticipating this shift should form part of the pre-procedural discussion with every patient presenting for Yonsei point injection.

The Yonsei point, validated cadaverically by Hwang et al. [5] and confirmed in a randomised trial by Gong et al. to be equivalent in efficacy to direct LLSAN injection at 4, 12, and 24 weeks post-treatment [6], remains the most widely adopted single-site target in the management of gummy smile with BoNT-A. Meta-analytic data from Zengiski et al. quantify a mean gingival exposure reduction of 3.42 mm at two weeks, with effects declining towards baseline by 24 weeks [7], consistent with the systematic review of Rojo-Sanchis et al., which reported reductions of up to 6 mm at 12 weeks across broader muscle protocols [12]. Reported per-side doses at the Yonsei point range from 1.25 to 7.5 U; the 2 U chosen here reflected moderate EGD severity and the patient's relatively lean facial musculature [12,13].

The orbicularis oris encircles the oral aperture and, through its superior fibres, exerts a sphincteric inward force on the upper lip margin. When this force is not adequately counterbalanced, either constitutionally or as a consequence of elevator muscle relaxation, the vermilion inverts. Targeted partial relaxation of these fibres with small BoNT-A doses everts the lip passively without compromising functional seal [8].

A systematic review by Pitchford et al. found that 4-6 U distributed along the superior orbicularis consistently produced vermilion eversion with high patient satisfaction and only mild, self-resolving functional effects such as transient difficulty using a straw [8]. Germani et al. corroborated this in a prospective study of 17 women receiving 4 U of onabotulinumtoxinA, documenting a significant increase in upper lip height by stereophotogrammetry at 15 days without volumetric change and high patient-reported scores [11]. The 2 U used in the present case was deliberately conservative, accounting for the partial orbicularis tone shift already produced by the Yonsei point injections.

**Figure 3:**
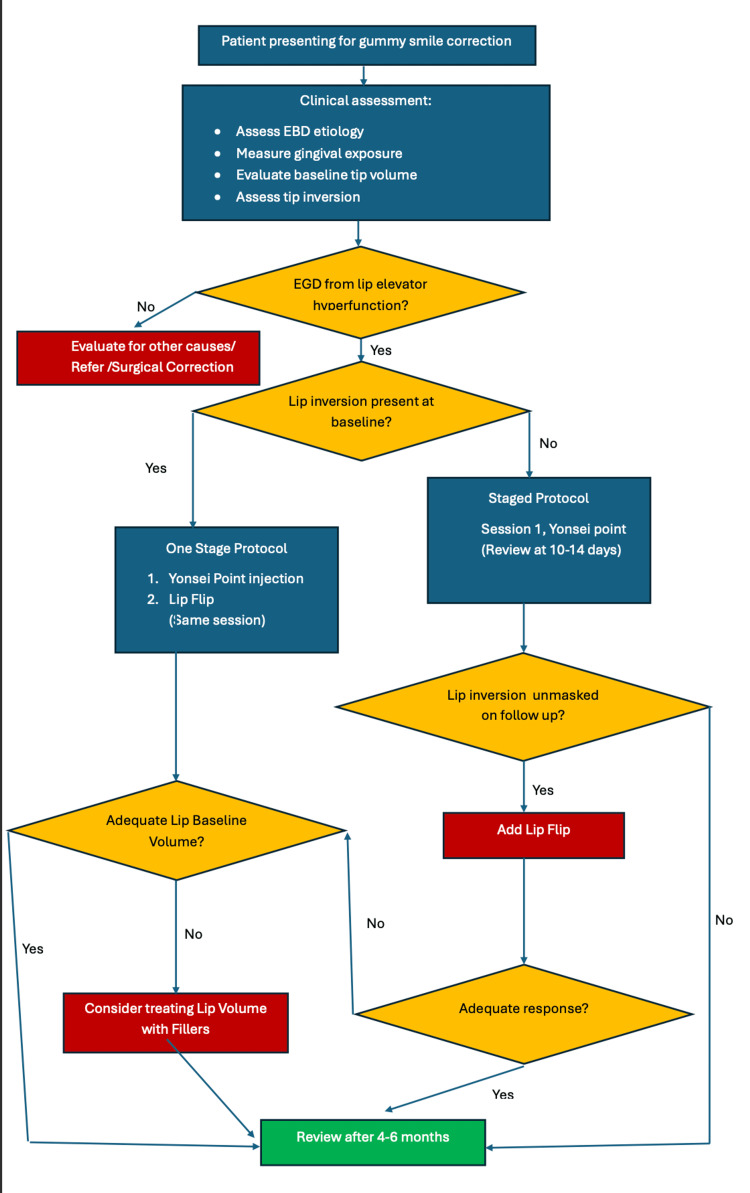
Management algorithm for gummy smile with concomitant lip inversion Patients with lip elevator hyperfunction as the dominant mechanism are assessed for lip inversion at baseline. When identified at the first consultation, a one-stage combined protocol (Yonsei point plus lip flip on the same day) is preferred. When inversion is unmasked at a 10–14-day review, a staged lip flip is added at that visit. Baseline lip volume guides the choice between neuromodulation alone and neuromodulation combined with hyaluronic acid filler. Both components require re-treatment at approximately 4–6 months. EGD = excessive gingival display; U = units Image created by the authors using Microsoft Word (Microsoft Corporation, Redmond, Washington).

When lip inversion is evident at the first consultation, a one-stage protocol of Yonsei point injection combined with low-dose lip flip on the same day avoids the inconvenience of a return visit. The staged approach used here, though necessitated by the unmasking of inversion at follow-up, achieved an equally satisfactory result and may sometimes be preferable, since the contribution of the orbicularis to lip dynamics cannot always be predicted prior to elevator relaxation (Figure 3).

Lip inversion in EGD patients can present in distinct ways that have practical implications for the timing and sequencing of treatment. Some patients display a clear vermilion inversion at rest and during smiling before any intervention, making the combined approach appropriate at the outset. Others, as in this case, exhibit only subtle inversion at baseline, which becomes the dominant finding once gingival exposure is corrected. A third group may develop apparent inversion as an unintended consequence of excessive Yonsei point dosing, in which the elevator muscles are over-relaxed and the orbicularis acts relatively unopposed. Clinicians should assess for all three possibilities: examining the lip contour during maximal smile before treatment, tempering Yonsei point doses in lean-muscled patients, and scheduling a review at 10-14 days specifically to detect post-correction inversion.

In patients with thin or age-attenuated lips where vermilion show is constitutionally limited, lip eversion alone, even when technically successful, may be insufficient, and hyaluronic acid augmentation provides the added volume necessary for a satisfactory result [4,9]. This was not required here because resting lip volume was adequate and eversion was all that was needed. This distinction is essential, as recommending filler to a patient with good lip volume and pure inversion adds procedural complexity, cost, and the risks attendant on volumising injections.

## Conclusions

Lip inversion should be sought as a routine part of every EGD assessment, not only because it may coexist with gingival display but also because its prominence can increase following elevator correction. Where it is identified before treatment, a combined Yonsei point plus lip flip protocol on the same day is practical and avoids an additional visit. Where it is unmasked at follow-up, a staged lip flip, as illustrated here, resolves the finding without necessitating dose escalation or filler. Patient selection should factor in baseline lip volume: those with adequate volume gain satisfactory results from neuromodulation alone, while those with thin lips may still require augmentation. Prospective studies comparing concurrent and staged protocols would help refine dosing thresholds and candidate selection for this combined approach.
